# Application of atomic force microscopy in morphological observation of antisense probe labeled with magnetism

**Published:** 2008-01-23

**Authors:** Ming Wen, Bibo Li, Wei Bai, Shaolin Li, Xueheng Yang

**Affiliations:** 1 Department of Radiology, The First Affiliated Hospital; 2 Department of Biopharmaceutical, Pharmacy College; 3 Department of Radiation Oncology, Basis Medicine College, Chongqing Medical University, Chongqing, China; 4 Hengrui Nanotechnology Workstation, Chongqing University, Chongqing, China

## Abstract

**Purpose:**

To explore the possibility of the c-erbB2 oncogene antisense probe labeled with superparamagnetic iron oxide (SPIO) nanoparticles as a target contrast agent for magnetic resonance (MR) imaging whose morphology was observed with atomic force microscopy (AFM), and its efficiency was examined by MR imaging.

**Methods:**

The c-erbB2 oncogene antisense probe labeled with SPIO was synthesized by a chemical cross-linking approach. Its morphology was observed with AFM.

**Results:**

The chemical constitution of c-erbB2 oncogene antisense probes can be observed with AFM. The molecular structure of probes is easily visualized under AFM. Probes with diameters of 25–40 nm are in order, follow uniformity and the arrangement rule, can be separated from each other, and appear as cubes with a rugged surface morphology. Strong, low signals of the probes in transfected cells were observed by MR cellular imaging.

**Conclusions:**

AFM is ideal for morphological observation and for analyzing the molecular structure of synthesized c-erbB2 oncogene antisense probes.

## Introduction

Malignant tumors are common and occur frequently in human diseases. The key to increasing the survival rate and improving quality of life is to diagnose malignant tumors at an early stage. Early visualized detection of oncogene expression provides novel early treatment of tumors through the antisense oligodeoxynucleotide (ASODN), which regulates gene expression by preventing genetic information from transferring to the protein. The development of molecular imaging at the end of the 20th century has provided a novel visualization for early and non-invasive diagnoses of diseases. In research on molecular imaging, target probes with good specificity and high affinity are critical factors to the success of in vivo molecular imaging [1,2]. The diameter of probe nanoparticles directly influences its distribution in a living body. In the present study, we prepared the ASODN of complementary c-erbB2 oncogene using the gene synthesis technique and labeled the superparamagnetic iron oxide (SPIO) nanometer using the chemical cross-linking method to produce the antisense probe. With atomic force microscopy (AFM), we observed the morphology of antisense probes labeled with SPIO to explore its potential as a target contrast agent for magnetic resonance (MR) imaging.

## Methods

### Main materials and apparatus

SPIO nanoparticles coated with glucan (20–35 nm) were prepared by our task group. Its core is Fe_3_O_4_ crystal with a diameter of 5 nm [3]. Oncogene c-erbB2 ASODN was synthesized by Shanghai Shenggong Biotechnology Co., Ltd. (Shanghai, China); its sequence is 5′-CTC CAT GGT GCT CAC-3′. Sodium periodate, sodium borohydride, and sodium bicarbonate were domestic analytical reagents. The DF-101B type homeothermic magnetic stirrer and 868 type pH-value measurement instrument were purchased from Thermo Orion Company (Waltham, MA). The HiprepTM16/60 type purification column was purchased from Amersham Bioscience Company (Uppsala, Sweden); the Vc60 type ultrasonic processor was purchased from Sonics & Materials Company, Inc. (Newtown, CT); and the IPC-208B type AFM was developed at the Hengrui Nanotechnology Workstation, Chongqing University (Chongqing, China).

### Preparation of antisense probe labeled with SPIO

At room temperature, the preparation of the c-erbB2 oncogene antisense probe was performed by using the chemical cross-linking approach [4,5]. First, 250 µl SPIO was added to 10 µl of 0.1 M sodium periodate, and after mixing, it was shaken at the rate of 200 revolutions/min for 30 min at 25 °C to produce oxidative SPIO. The oxidative SPIO was then dialyzed in 0.9% normal saline for 2 h then in 20 mM sodium bicarbonate for 1 h to produce a purified SPIO solution. ASODN (33 µg; 1 OD) was dissolved in 100 µl ultra pure water, and after adding purified SPIO, ASODN was shaken at the rate of 200 revolutions/min, for 24 h at 25 °C to produce the preliminary product of antisense probe. Sodium borohydride (20 µl of 1 M solution) was added into the preliminary product of antisense probe and shaken for 30 min to produce the end product of the antisense probe. Finally, the end product of antisense probe was purified with a high performance liquid gel column, sub-packed, and stored in a refrigerator at 4 °C for further use.

### Atomic force microscopy observation

The highest resolving capability of the IPC-208B type AFM is 0.1 nm of lateral way and 0.01 nm of longitudinal way, and its largest scanning area is 25 mm×25 mm. Small, flaky metal used as a carrier for observation was washed with acetone; after it dried, the antisense probe sample was placed in drops on the dry, flaky metal and then dried at room temperature. The surface morphology of the sample was observed in a large scale scanning area of 700 nm×700 nm and 1000×1000 pixels. The molecular structure and microstructure of the sample was observed in a small scale scanning area of 9 nm×9 nm and 800×800 pixels. The original image data were transmitted to a computer, and 3D reconstruction was performed with G2DR software.

### In vitro magnetic resonance imaging

SK-Br-3 oncocytes were cultured using 10% calf serum, 100 U/ml penicillin, and 100 µg/ml streptomycin at 37 °C in a CO_2_ incubator. A 2-ml ASODN probe with 25 µg/ml of Fe concentration was added to actively proliferate logarithmic growth phase cells at a concentration of 4×10^6^ cells/ml. The oncocytes were collected after the 12 h culture and washed twice with D-Hanks solution. The other oncocytes without probes were used as the control group. For in vitro MR imaging, transfected and untransfected cells with concentrations of 5×10^5^ cells/ml were placed into Ependorf tubes, which were fixed with three inches of surface coil. The probe culture medium (Fe; 25 μg/ml), the no-probe culture medium, and the distilled water were used as control samples. MR transection scanning was performed in all five samples by using a 1.5 T instrument (GE Company, Milwaukee, WI). The collecting sequence was GRE30° (TR/TE 27.3/1.7 ms) with the field of view as 16 cm, slice thickness of 5 mm, and a matrix of 256×128.

## Results

**Figure 1:**
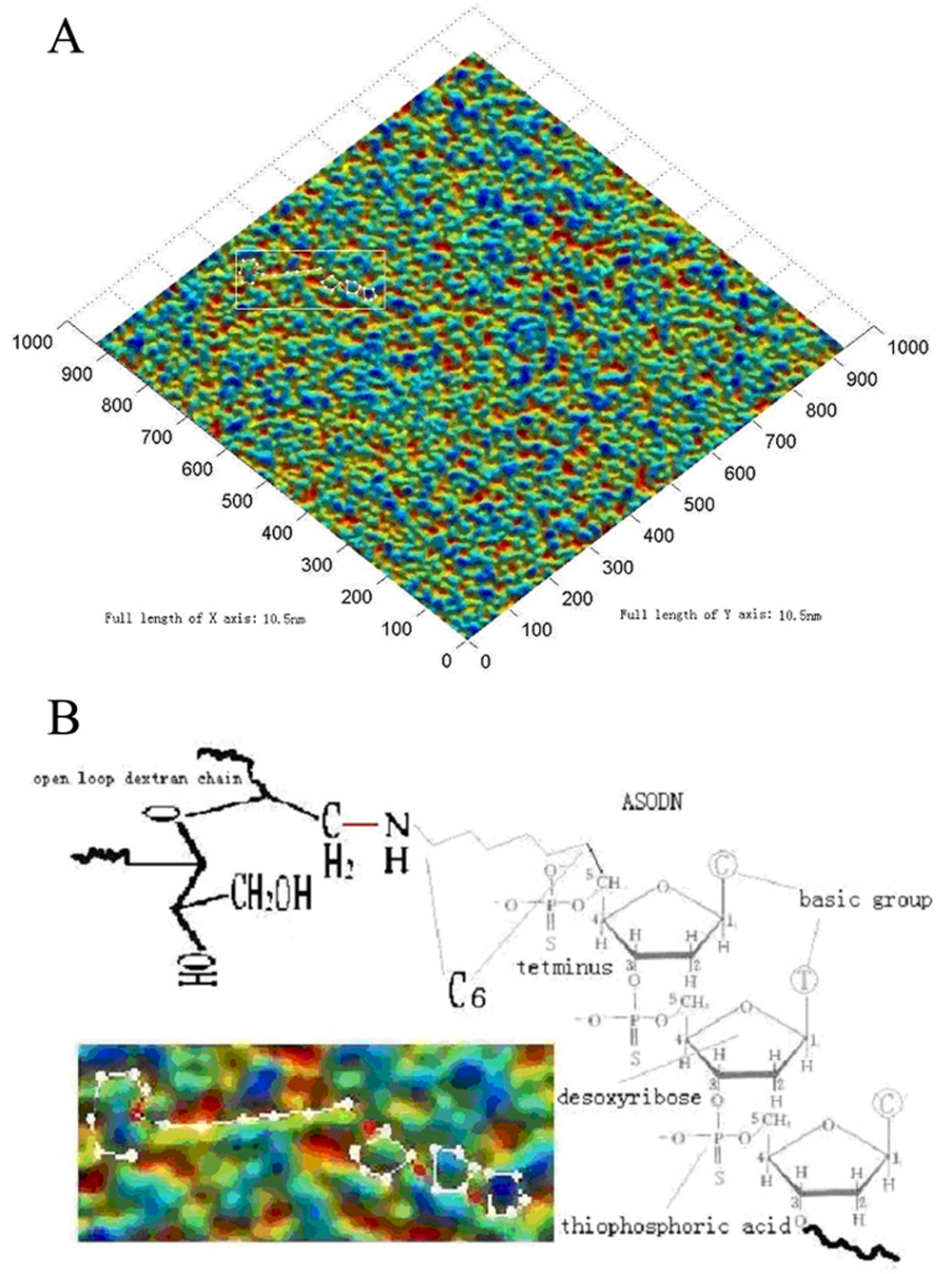
Color image of c-erbB2 oncogene antisense probe in the atomic force microscopy. **A**: Distribution of c-erbB2 oncogene antisense probe under atomic force microscopy is shown. The red, green, and blue colors in the image indicate probe density from the highest (red) to the lowest (blue). **B**: The structure for part of dextran and ASODN exhibited. The framed rectangle area in **A** shows part of the constitution of dextran and ASODN; the atom and basic groups were outline with white dots. The semicircle on the left was an open loop of dextran, the straight line in the middle was a carbochain, which linked amino-group and six carbon atoms of ASODN, and the three pent-loops on the right were deoxyribose loops at the end of ASODN. The total diameter of the antisense probe was 25–40 nm, and the length of ASODN was 5 nm.

**Figure 2:**
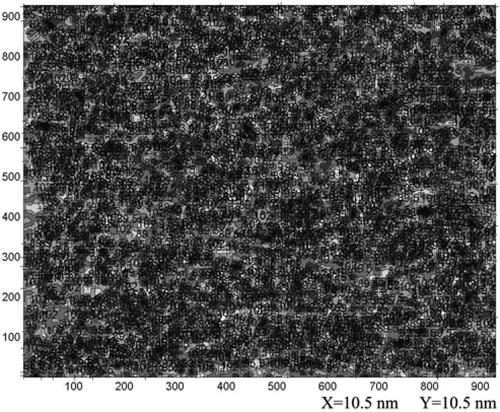
Elevation contour image of c-erbB2 oncogene antisense probe in the atomic force microscopy. The density of the c-erbB2 oncogene antisense probe was divided into 8 grades which are used to differentiate relative height, and the rugged surface morphology was marked with figures on image**.**

The original image was treated with Gaussian filtering, speckle filtering, dust filtering, and scratch filtering, and after regulating brightness and contrast, a color image of the c-erbB2 oncogene antisense probe (Figure 1) and elevation contour image (Figure 2) were obtained. Graduations on coordinate axes in all images are pixel values, and indication ranges of the X and Y directions are 350 nm with a total of 1000 pixel levels. Reds, greens, and blues in the color images reflected probe density. Red represented the highest density, green the second highest, and blue the lowest (Figure 1A). Based on the atom and basic groups observed with AFM, the structural relationship between dextran and ASODN is clearly visualized (Figure 1B). Dextran loops, carbochain linking amino group and six carbon atoms of ASODN, and deoxyribose loops were observed. Thus, ASODN is linked closely with dextran and are arranged in an orderly manner. The total diameter of the antisense probe was 25–40 nm, and the length of ASODN is 5 nm. In the elevation contour image, the density of the sample was divided into eight grades, which are used to differentiate relative height; the rugged surface morphology was marked with figures on the image.

Figure 1 and Figure 2 clearly display images of antisense probe nanoparticles that are in order, follow uniformity and the arrangement rule, can be separated from each other, and appear as a cube with a rugged surface morphology.

**Figure 3:**
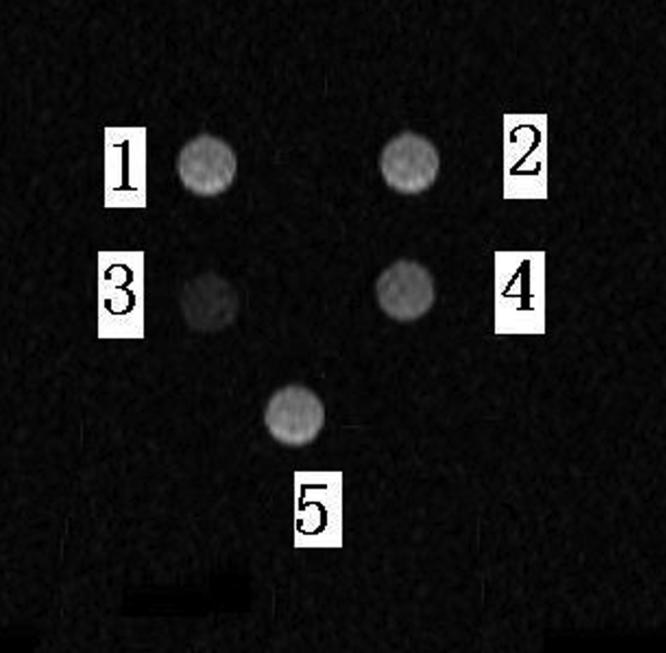
Magnetic resonance image of c-erbB2 antisense probe transfected SK-Br-3 cells. Transfected and untransfected cells, a probe culture medium, a no-probe culture medium, and distilled water were placed into Ependorf tubes and scanned using MR transactions. 1: untransfected cells, 2: no-probe culture medium, 3: transfected cells, 4: probe culture medium, 5: distilled water. From the figure, we can observe that c-erbB2 antisence probe can improve the magnetic properties of SK-Br-3 cells and decrease the signal intensity in MR scanning obviously.

To test the suitability of the c-erbB2 oncogene antisense probe for MR imaging, SK-Br-3 oncocytes were transfected with and without antisense probes. Transfected and untransfected cells, probe culture media, no-probe culture media, and distilled water were placed into Ependorf tubes and scanned by MR transactions. Untransfected cells, no-probe culture media, and distilled water distinctly showed a high signal. The group with distilled water had the highest signal, transfected cells and cells with probe culture media had distinctly low signals, and the transfected cells had the lowest signals (Figure 3). These results indicate that the c-erbB2 oncogene antisense probe is suitable for MR imaging.

## Discussion

In research on molecular imaging, molecular probes are usually classed into two types based on their diameters: general type (diameter >40 nm) and super-small type (diameter <40 nm). Researchers have been interested in super-small probes because they can avoid the clearance action of the reticuloendothelial system, increase in vivo retention time, and possess better target ability [6].

At present, the methods directly showing morphology of probes usually include X-ray diffraction analysis, scanning tunnel microscope, and AFM. In X-ray diffraction, crystal was irradiated with X-rays to produce scattering, superposition, or cancellation of each other between scattered waves. This resulted in diffraction phenomena, which reflect atomic distribution in the crystal so that the X-ray diffraction only determines the preferential growth surface and different components of the sample [7]. Using the tunnel effect principle of quantum mechanics, the rugged surface morphology of samples was detected with a scanning tunnel microscope through the changes of the current between the probe and a conductor surface, and its precision may be at the atomic scale. However, the process of treating the sample is complex. Samples generally require fixation, dehydration, dryness, and gilt that can readily conceal or destroy the fine structure of the sample surface. Since magnetic fields may interfere with electron beams emitted from a scanning tunnel microscope, our magnetic sample with 69.32875 emu/g Fe of saturation magnetization resulted in an unstable image with bad contrast and definition and in a diminished reliability of the test result [8].

AFM is one of the most important advances in the research of imaging technology of the last 10 years. In 1986, Gerd Binnig and Heinrich Rohrer again produced AFM following the successful development of the first scanning tunnel microscope. AFM obtains the information of sample surface morphology through the very fine movement of probe cantilever, resulting from changes in interaction force and tunnel current between the sample surface atom and probe point atom; its resolution may be on an atomic scale. Thus, imaging of a three-dimensioned surface structure of a sample may be formed directly in a physiologic solution, and dynamic and real-time observations for in vivo histomorphological variations under physiology and pathological conditions can be performed. Compared with X-ray diffraction and scanning tunnel microscopes, the sample treatment time is short, the procedures are simple, and the sample quantity and cost required are small, making AFM an ideal microscope for observing the morphology of a molecular probe [8,9].

There have been few reports on the observation of morphology of the c-erbB2 oncogene antisense probe with AFM at home and abroad. At present, the resolving range of commercialized AFM is between 1 nm and 100 nm, possessing four freedom degrees [10]. Our IPC-208B type AFM possesses 13 freedom degrees with 25 mm×25 mm as the largest scanning area. Its highest resolving capability is 0.1 nm of lateral way and 0.01 nm of longitudinal way, which greatly enhances the measuring and processing range [9]. In our experiment, ASODN was labeled with SPIO using the chemical cross-linking approach because the coating bacterial of SPIO, the hydroxide radical of glucan, is oxidated to produce an aldehyde group. This reacts with ASODN to form a Schiff base, leading to a covalent union [4,5] (PAGE and gel filtration chromatography analysis indicated 92.5% of the cross-linking rate of the synthesized probe, which is reported in another paper). Thus, not only is the stability of the probe enhanced, but also the diameter of the nanoparticles is kept smaller to make it pass easily through gaps of vessels and tissue to get to the target cells. The surface morphology of the antisense probe can be seen in images obtained with AFM scans; the diameter of the probe is 25–40 nm, which suggests the synthesized probes possess characteristics to be a targeting contrast agent for MR imaging. Finally, based on our observations, we conclude that the synthesized c-erbB2 oncogene antisense probes are suitable for MR imaging.
